# Supplementary material for: HIVprotI: an integrated web based platform for prediction and design of HIV proteins inhibitors
Source: J Cheminform. 2018 Mar 9;10:12. doi: 10.1186/s13321-018-0266-y (PMC5845081; doi:10.1186/s13321-018-0266-y)
Supplement: Supplementary file 2 — Additional file 2. Source code of HIVProtI web server. [file 13321_2018_266_MOESM2_ESM.zip › HIVprotI_Source-code/hivprotI/weka-3-6-6/doc/weka/core/RandomVariates.html]

RandomVariates


---


|  |  |  |
| --- | --- | --- |
| **Overview** \| **Package** \| **Class** \| **Tree** \| **Deprecated** \| **Index** \| **Help** \| **Weka's home** | |  |
| **PREV CLASS**   **NEXT CLASS** | **FRAMES**    **NO FRAMES**     **All Classes** |  |
| SUMMARY: NESTED \| FIELD \| CONSTR \| METHOD | DETAIL: FIELD \| CONSTR \| METHOD |  |


---


## weka.core Class RandomVariates

```
java.lang.Object
  java.util.Random
      weka.core.RandomVariates
```

**All Implemented Interfaces:**: java.io.Serializable, RevisionHandler

---

``` public final class RandomVariates extends java.util.Random implements RevisionHandler ```

Class implementing some simple random variates generator.

**Version:**
:   $Revision: 5360 $

**Author:**
:   Xin Xu (xx5@cs.waikato.ac.nz)

**See Also:**: Serialized Form

---

**Constructor Summary**

`RandomVariates()`
  
          Simply the constructor of super class

`RandomVariates(long seed)`
  
          Simply the constructor of super class


| **Method Summary** | |
| --- | --- |
| `java.lang.String` | `getRevision()`             Returns the revision string. |
| `static void` | `main(java.lang.String[] ops)`             Main method for testing this class. |
| `double` | `nextErlang(int a)`             Generate a value of a variate following standard Erlang distribution. |
| `double` | `nextExponential()`             Generate a value of a variate following standard exponential distribution using simple inverse method. |
| `double` | `nextGamma(double a)`             Generate a value of a variate following standard Gamma distribution with shape parameter a. |

**Methods inherited from class java.util.Random**

`nextBoolean, nextBytes, nextDouble, nextFloat, nextGaussian, nextInt, nextInt, nextLong, setSeed`

**Methods inherited from class java.lang.Object**

`equals, getClass, hashCode, notify, notifyAll, toString, wait, wait, wait`

**Constructor Detail**

### RandomVariates

```
public RandomVariates()
```

:   Simply the constructor of super class

---


### RandomVariates

```
public RandomVariates(long seed)
```

:   Simply the constructor of super class

    **Parameters:**: `seed` - the seed in this random object


**Method Detail**

### nextExponential

```
public double nextExponential()
```

:   Generate a value of a variate following standard exponential
    distribution using simple inverse method.

    Variates related to standard Exponential can be generated using simple
    transformations.

    :   **Returns:**: a value of the variate

---


### nextErlang

```
public double nextErlang(int a)
                  throws java.lang.Exception
```

:   Generate a value of a variate following standard Erlang distribution.
    It can be used when the shape parameter is an integer and not too large,
    say, <100. When the parameter is not an integer (which is often named
    Gamma distribution) or is large, use `nextGamma(double a)`
    instead.

    :   **Parameters:**: `a` - the shape parameter, must be no less than 1 **Returns:**: a value of the variate **Throws:**: `java.lang.Exception` - if parameter less than 1

---


### nextGamma

```
public double nextGamma(double a)
                 throws java.lang.Exception
```

:   Generate a value of a variate following standard Gamma distribution
    with shape parameter a.

    If a>1, it uses a rejection method developed by Minh(1988)"Generating
    Gamma Variates", ACM Trans. on Math. Software, Vol.14, No.3, pp261-266.
      
    If a<1, it uses the algorithm "GS" developed by Ahrens and Dieter(1974)
    "COMPUTER METHODS FOR SAMPLING FROM GAMMA, BETA, POISSON AND BINOMIAL
    DISTRIBUTIONS", COMPUTING, 12 (1974), pp223-246, and further implemented
    in Fortran by Ahrens, Kohrt and Dieter(1983) "Algorithm 599: sampling
    from Gamma and Poisson distributions", ACM Trans. on Math. Software,
    Vol.9 No.2, pp255-257.

    Variates related to standard Gamma can be generated using simple
    transformations.

    :   **Parameters:**: `a` - the shape parameter, must be greater than 1 **Returns:**: a value of the variate **Throws:**: `java.lang.Exception` - if parameter not greater than 1

---


### getRevision

```
public java.lang.String getRevision()
```

:   Returns the revision string.

    :   **Specified by:**: `getRevision` in interface `RevisionHandler`
    :   **Returns:**: the revision

---


### main

```
public static void main(java.lang.String[] ops)
```

:   Main method for testing this class.

    :   **Parameters:**: `ops` - # of variates/seed, default is 10/45


---


|  |  |  |
| --- | --- | --- |
| **Overview** \| **Package** \| **Class** \| **Tree** \| **Deprecated** \| **Index** \| **Help** \| **Weka's home** | |  |
| **PREV CLASS**   **NEXT CLASS** | **FRAMES**    **NO FRAMES**     **All Classes** |  |
| SUMMARY: NESTED \| FIELD \| CONSTR \| METHOD | DETAIL: FIELD \| CONSTR \| METHOD |  |


---
